# Conditions Underlying the Appearance of Spontaneous Otoacoustic Emissions in Mammals

**DOI:** 10.1007/s10162-024-00950-5

**Published:** 2024-05-17

**Authors:** Geoffrey A. Manley

**Affiliations:** Cochlear and Auditory Brainstem Physiology, Department of Neuroscience, School of Medicine and Health Sciences, Cluster of Excellence “Hearing4all”, Carl von Ossietzky University Oldenburg, 26129 Oldenburg, Germany

**Keywords:** Otoacoustic emission, Coupling, Tectorial membrane, Space constant

## Abstract

Across the wide range of land vertebrate species, spontaneous otoacoustic emissions (SOAE) are common, but not always found. The reasons for the differences between species of the various groups in their emission patterns are often not well understood, particularly within mammals. This review examines the question as to what determines in mammals whether SOAE are emitted or not, and suggests that the coupling between hair-cell regions diminishes when the space constant of frequency distribution becomes larger. The reduced coupling is assumed to result in a greater likelihood of SOAE being emitted.

## Introduction

Beginnings and endings are times for reflection, and the passing of colleagues offers opportunities to analyse the progress of a scientific field during their lifetime. After a productive career studying various types of otoacoustic emissions in different vertebrate species, Glenis Long’s approach is epitomized by the title of her 2011 abstract: *Why bother looking at a range of species?* Her idea was to derive a foundation of data and theory, mainly from different mammalian species, that would enable a much better understanding of human hearing. To quote Glenis: “This paper will provide evidence from psychoacoustic, evoked potential and OAE research that comparative research is essential if we are to fully understand human auditory processing.” [1]. Since I also have spent decades looking at a broad range of species - mostly, however, of non-mammals – these are obviously kindred ideas to my own.

Since their ears have a common origin, it is appropriate to combine knowledge derived from all land vertebrates and examine features of their hearing organs that enable them all to produce particular and often unique, patterns of otoacoustic emissions. Here, the emphasis is on spontaneous emissions (SOAE), which provide a unique window into inner-ear function. SOAE are not, however, measurable in all species, being fairly uncommon in mammals. They are very common in advanced amphibians [2, 3], and have been reported in all lizard species so far examined [4]. In birds, they have only been reported in the barn owl [5]. Since cochlear structure is likely to strongly influence the spectral patterns of SOAE, this review is restricted to mammals, in the expectation that in general, structural coupling will be similar in all mammalian species, but will differ from that of non-mammals. This is supported, for example, by the fact that about 50% of the tectorial membrane of mammals consists of several types of the elastic protein collagen [6], that is absent from the tectorial membranes of birds [7] and probably from those of other non-mammals. This profound difference in the tectorial membranes make it very likely that between mammals and non-mammals differences in the strength of the coupling provided by the TM will influence the patterns of interactions between hair cells and thus the patterns in emissions.

The review examines the question as to why SOAE are fairly uncommon in mammals, and initial ideas as to what could determine whether the conditions for SOAE generation exist or not. In doing so, I do not provide a comprehensive review, but have chosen in each case examples that illustrate patterns. The most important idea is based on what I have termed the space constant, a value that describes the scale underlying the mapping of frequency along the cochlea. The space constant is the length of cochlear epithelium that is occupied by one octave. Although some great exceptions have been described (referred to below as “auditory foveae”), and few cochleae have space constants that are precisely the same along the whole cochlea, in most cases, the deviations are small. In humans, for example, using critical-band measurements, Greenwood [8] speculated that at least above 1 kHz, each higher octave occupies the same space along the basilar membrane – the distribution of frequencies is exponential. Numerous studies since then have shown this to be close to the truth in many species of mammals [e.g. 9–12], birds [13] and lizards [14, 15]. Given the assumption of an exponential distribution and knowing the length of the basilar membrane and the frequency limits of hearing, an approximate “space constant” for each species can be calculated.

## Where are SOAE Found and What are Their Peculiarities in Different Vertebrate Groups?

To date, SOAE have been reported from all major groups of land vertebrates: amphibians, reptiles, birds, and mammals. The hearing organs of these groups differ, sometimes radically, in their structure, leading to the expectation that emission patterns produced would also differ in particular ways and these differences might provide clues to the origins of SOAE. Some features of SOAE are immediately obvious, e.g., the largest hearing organs less commonly produce SOAE. Thus, the very small (sometimes less than 0.2 mm) organs of lizards are highly reliable SOAE generators [4, 16], as is the case in advanced amphibian hearing organs [3, 17], whereas the number of mammalian and avian species known to produce SOAE is a much smaller proportion. There is little doubt that active processes are the substrate of sensitivity in all vertebrate hearing organs [16, 18], and thus that all species have the potential of emitting SOAE, so what determines whether they can be measured or not?

## Amphibians

**Fig. 1:**
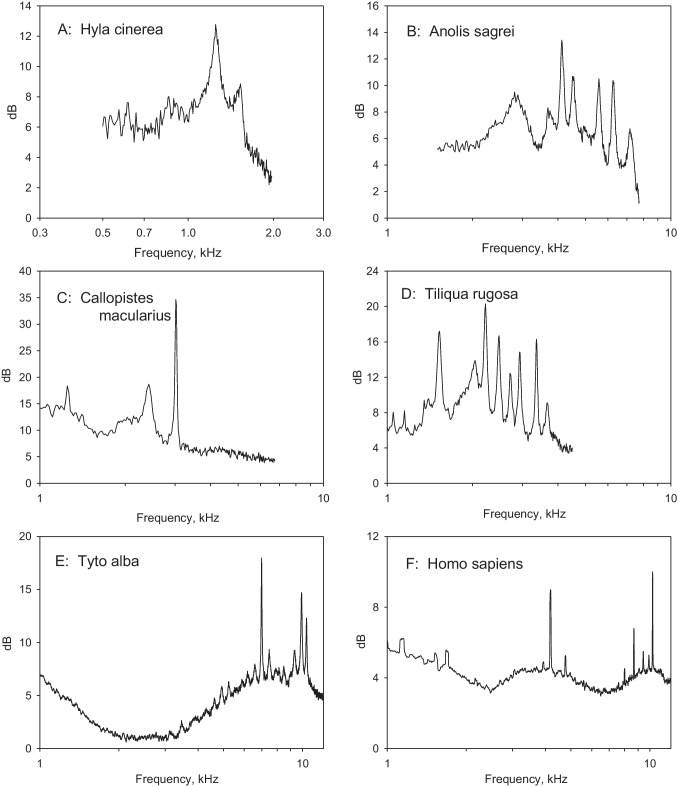
Individual ear-canal spectra in quiet from six different species of land vertebrates, an anuran amphibian (**A**), three lizard species with differing tectorial-membrane structure (**B** to **D**, see text), a bird (**E**), and a human (**F**) representing mammals. Note that the frequency scales are not uniform, and all dB level axes are scaled relative to an artificial level of 0dB. The peaks below 2kHz in F are likely to be artefacts

Most studies of amphibian hearing were made on anurans, advanced amphibians that are mostly known as frogs and toads. While there are structural differences between taxonomic groups, the hearing organ generally consists of two physically separated hair-cell areas, the amphibian and the basilar papilla (the latter may be homologous with the basilar papilla of other taxa, and thus with the organ of Corti of mammals). The amphibian papilla responds to low- and mid-frequencies (approx. 0.1 to 1.5 kHz), the basilar papilla to higher frequencies (approx. 2.5 to 4 kHz), these ranges being separated and species-specific [19]. SOAE in amphibians were some of the earliest described and are generally found in “advanced” anurans (those with a tympanic middle ear and a well-developed amphibian papilla). They show peak frequencies between 0.645 and 1.68 kHz [19]. SOAE occur mostly in the form of two wide peaks of amplitude up to 13 dB above the noise level, as reported, e.g., in the edible frog *Rana esculenta* [17] and in a tree frog *Hyla cinerea* [19] (Fig. 1A). The frequencies of the peaks (near 0.8 and 1.2 kHz in *Rana*, 0.85 and 1.5 kHz in *Hyla*) more closely correspond to those represented in the amphibian, but not the smaller basilar papilla. Van Dijk et al. [3] emphasized, however, that the frequency range of SOAE does not correspond exactly to the ranges of either auditory papilla. Since, however, both SOAE peak frequencies and neural tuning are temperature sensitive, this inconsistency needs further study.

## Reptiles

There are no reports of SOAE from the ears of chelonians (tortoises and turtles), which generally only hear below 1 kHz [2], but there have been (unpublished and unsuccessful) attempts to measure them. To date, there are unfortunately no reports of SOAE studies in crocodilians (alligators, crocodiles, garials), thus we have no information from this group. In contrast, more than 30 lizard species have been examined [20, 21], in some cases in great detail, revealing SOAE in every single species. In all cases, the centre frequency of each spectral peak was temperature dependent, a behaviour also seen in lizard auditory-nerve fibres [2]. There are characteristic, partly family-specific, variations in the anatomy of lizard auditory papillae, and these are reflected in the patterns of the SOAE, providing interesting clues as to the mechanisms determining the SOAE spectral configurations in lizards, in which hair-cell coupling plays an important role [22]. Here, only two anatomical features relevant to SOAE patterns will be considered. First, all lizard papillae are divided into a low-frequency hair-cell area (responses below ~ 1 kHz) and a high-frequency area (above 1 kHz). Second, tectorial-membrane structure varies between lizard families. While the low-frequency area is always covered by a single – sometimes very large – mass of tectorial material, the tectorial coupling provided for the high-frequency area varies from absent (in, e.g., iguanids, agamids, and anguids), to a chain of “sallets” (in, e.g., skinks, geckos) and a continuous tectorial membrane (in, e.g., varanids, teiids).

The low-frequency area has never been observed to produce SOAE, which are thus confined to the higher frequencies (thus from ~ 1 kHz generally up to 5–8 kHz). This is consistent with the lack of SOAE from the – only low-frequency – chelonian papilla. The structural variation in tectorial material in lizard families correlates, at least in a general way, with the spectral patterns of SOAE [15]. In the absence of a tectorium, a condition that is only observed in very small papillae, SOAE spectral peaks occur even in large numbers – up to ~ 14 – are generally relatively small, and lie between 1.0 and 8.0 kHz, Fig. 1B). When the tectorium is continuous, the number of peaks is smaller, sometimes only 2 or 3 large peaks were found (e.g., in teiids [23], or varanids [24], Fig. 1C). Interestingly, in the presence of sallets, which are also only found in larger papillae, the spectra resemble those seen in the absence of a tectorial membrane, i.e. many, generally small, spectral peaks (Fig. 1D). Sallets are only linked to their immediate neighbours by thin connections, suggesting that longitudinal coupling is poor. The tectorial membrane not only stabilizes the peak frequencies of SOAE (presumably through the physical coupling of nearby hair cells), but also influences the spectral patterns.

The underlying reasons for the various spectral patterns in lizards have been explained as follows [25, 26]: In very small papillae, the same high-frequency range as in other, often much larger, lizard papillae is confined to a small length of papilla, e.g., from 1.0 to 5.0 kHz within 0.2 mm, corresponding to less than 100 µm, or less than 10 hair-cell rows, per octave. Thus, neighbouring hair cells will differ in their best frequency response by about one-tenth of an octave. A wide-ranging coupling of neighbouring hair cells would hugely decrease any ability to respond differentially to small differences in frequency, and thus greatly limit the frequency selectivity. Fairly sharp frequency selectivity has been reported in auditory nerve fibres of these species [20, 21], and in suppression-tuning curves of their SOAE [20, 21], a selectivity that is only possible due to the lack of coupling in the absence of a tectorial membrane. Sallets found in much longer papillae fulfil a similar function. They do couple hair cells, of course, but due to the longer papillae (covering the same frequency range as in the tiny papillae), the frequency range of each set of coupled hair cells is narrow. In fact, in geckos often only a single row of hair cells across the papilla (all presumably having the same best frequency) is coupled by any given sallet [27]. In skinks, 2 or 3 rows of hair cells are united under each sallet [22]. This system not only does not reduce frequency selectivity, through local coupling it actually deepens the tuning selectivity curves of auditory-nerve fibres and of suppression tuning curves of SOAE, as demonstrated for SOAE in the Bobtail skink [28]. In stark contrast to the above, in species with a continuous tectorial membrane, there are generally fewer and larger peaks in the spectrum, suggesting that the coupling efficiency along a greater length of the papilla is strong, and this reduces any frequency discrimination based on micromechanical tuning [21].

## Birds

SOAE have only been reported from one species of bird, the barn owl, although starlings and chickens have also been studied [17]. The barn owl is an auditory specialist, with several features that hugely increase its auditory efficiency: e.g., its facial ruff increases its threshold sensitivity by about 20 dB [26]. Not only is its cochlea very long for a bird (12 mm), but fully half of the auditory papilla is a fovea specialized for processing only frequencies within the highest octave, from 5 to 10 kHz [29]. The barn owl also produces a wide spectrum of smaller and larger SOAE peaks [30]. Despite the highly non-exponential distribution of frequency on the papilla due to the fovea [31], the frequency intervals between the peaks were remarkably similar (~ 420 Hz) across the entire spectrum [5, 19] (Fig. 1E). This distribution pattern remains unexplained, but implies cochlear-scale interactions among hair cells. Nothing similar has been observed in any mammal.

## Mammals

Among mammals, SOAE have been reported from humans, macaque monkeys, chinchillas, guinea pigs, and two mutant mouse strains. Based on cochlear length, in which most mammals have longer – often much longer – cochleae than non-mammals, a higher incidence of SOAE – with a wide range of peak frequencies as in humans - might be expected. Instead, there is mostly little data, and more careful examination of ear-canal spectra in quiet are needed. Here, I will not consider the few cases of very loud spontaneous emissions in humans and animals (e.g., in a dog, [32]), which are likely due to aberrant cochlear anatomy. In chinchillas, two reports each described only two SOAE peaks, but these were generally only after noise exposure [33, 34]. However, Long et al. [35] found a large number of SOAE in 60% of their chinchillas. In Macaque monkeys, fewer than 10% of animals showed SOAE [36]. Ohyama et al. [37] found SOAE below 2.3 kHz in 26% of guinea pig ears. By contrast, the percentages of occurrence in humans are much higher. Abdala et al. [38] reported SOAE in 85% of newborns, in 51%–68% of young adults and in 40% of the elderly (> 59 years). The observed sound-pressure levels of the SOAE in humans also decreased with time, and newborn children had SOAE that were 5 to 6 dB higher in level than those from young-adult ears and much higher than those in the elderly. Working with Talmadge, Murphy and Tubis, Glennis Long [39] reported SOAE in 72% of human adults, but in only 56% of ears. In 76 young adults, Penner et al. [40] found SOAE in 75% of female individuals and 58% of males, although no differences in SOAE spectral patterns between the sexes were reported. A typical spectrum from a young human is shown in Fig. 1F [41]. Thus, even among mammals there are substantial variations in how often SOAE occur, and these variations are not trivial to explain.

## Generating SOAE

How do the above data fit together, and what do they tell us about SOAE patterns? While on the one hand these demonstrate that while the title “Salient features of otoacoustic emissions are common across tetrapod groups and suggest shared properties of generation mechanisms” [30] emphasizes the generality of generation mechanisms, there are substantial unexplained issues. If SOAE generation is expected to be universally possible, why do many mammals not manifest such emissions? This opens the general question of this review – what are the conditions necessary for the expression or appearance of SOAE in ear-canal spectra? Since at least in all species of lizards, and possibly in all anuran amphibians [3], there is virtually a 100% guarantee that SOAE will be found, and in birds we can only work with data from one specialist species, the current discussion will be confined to mammals, but using the knowledge gained from all species. The clear influence of coupling on SOAE patterns in lizard papillae is a good starting point for a discussion.

The study of coupling in lizard papillae was carried out in, for example, models of the SOAE patterns in the Bobtail skink. This species has a large (for lizards) papilla (~ 2 mm, with ~ 2000 hair cells) in which the higher-frequency (> 1 kHz) hair cells are covered by tectorial sallets. Each sallet couples a local group of 10–25 hair cells [22, 28], and is itself only joined to its salletal neighbours through a thin connector. In their SOAE model, Vilfan and Duke [42] conclude that their coupling model mimics the behaviour observed in the living animal and that “… the varying properties of emissions amongst species are a consequence of different physical coupling between hair cells”. Extending that model, Wit et al. [43] found that “The most remarkable result is that the actual number of oscillating elements hardly influences the spectral pattern, explaining why spectra from very different papillar dimensions are similar. Furthermore, the spacing between spectral peaks primarily depends on the reactive coupling between the oscillator elements.” In a model of a different species that also has a large cochlea with salletal coupling (the Tokay gecko), Gelfand et al. [44] concluded that “…elastic coupling between oscillators … explains several properties of the spontaneous otoacoustic emissions in the gecko”.

There is little doubt that in all groups, hair-cell activity underlies SOAE production. Especially relevant here are the similarities in SOAE features, including details of their suppression tuning curves and of neural tuning. Thus, it is assumed that the energy underlying the sound output at the hair-cell level is always there – but why is some of it only “released” into the outside world in some species and at some frequencies rather than being equally obvious in all species and ears? One important clue was provided by work on two mutant strains of mice. Although control, wild-type (WT) mice have only once been reported to produce SOAE, two knockout mice strains frequently emit SOAE - the Ceacam16 knockout mouse that has no Henson’s stripe, and the Otoa knockout, in which the tectorial membrane is not attached to the spiral limbus, there is no Henson’s stripe, and the marginal band is disrupted [45, 46]. Both mouse mutant strains showed SOAE in most cases (e.g., 79% of Otoa mutants), although the frequencies of the peaks were clustered in different regions (Otoa mostly below 10 kHz, Ceacam mostly above 15 kHz). The authors assume that the disruptions to hair-cell coupling caused by the abnormal tectorial membranes are responsible for the dramatically increased numbers of SOAE. Is this an indication that strong coupling through the tectorial membrane can prevent SOAE? What if the same coupling strength is present in species where the frequency regions are much further apart – that have, in other words, a greater space or frequency-mapping constant?

Together with the influence of the various types of tectorial membrane on SOAE in lizards, this mouse data suggests that hair-cell coupling - mainly through the tectorial material - plays a critically important role in permitting the emergence of SOAE and patterning of these emissions. This can also be expressed in a more general form: coupling strength is the key element in the expression of SOAE peaks. While there are no iguanid, agamid or anguid lizard species that have a tectorial membrane over their high-frequency hair cells, and thus the “natural experiment” to observe SOAE or their absence in such cases does not exist, the argument that the lack of coupling is essential to preserve frequency discrimination in such tiny papillae is convincing. Where strong coupling is present in other families of lizards (e.g., varanids, teiids), there are only few, wide SOAE peaks of high amplitude, suggesting that a much wider range of frequencies and many more hair cells contribute to each peak than in iguanids, etc. [47]. The parallel to mutant mice is clear: Strong coupling reduces the occurrence of SOAE peaks, even in many mammals - in many cases to zero. Mutant uncoupling “releases” hair-cell groups from this constraint.

Together with the extensive literature on coupling in human cochleae (for refs see [40]), it is thus fair to conclude that coupling of hair cells underlies the grouping of sound energy seen in the peaks of human ear-canal spectra in quiet. Nonetheless, this does not explain differences between mammals – why are SOAE so common in humans, yet much less common, and generally missing, in some typical mammalian laboratory species? One aspect of coupling that has as yet received little attention is the concept of the space- or frequency-mapping constant. The space constant describes the length of hearing-organ available for the analysis of one octave of their species’ frequency range, and therefore is an indication of the physical distance between hair cells responding to different frequency ranges. Obviously, there are large differences in space constants even within lizards, since the total frequency response range is similar in all cases, but papillar length varies up to more than a factor of ten. Compared again to birds (total cochlear length up to 12 mm) and mammals (total cochlear length up to 60 mm), lizard space constants are tiny, yet frequency tuning at the level of the auditory nerve is comparable [47, 48]. This can only be possible by changing the coupling between hair cells or groups of hair cells, with much weaker coupling in shorter papillae. Within mammals, cochlear length also varies almost ten-fold (mouse 7 mm; elephant 60 mm) [49, 50], but the auditory ranges also vary, making a comparison of coupling only possible if species’ frequency range details are taken into account. Of course, there are different ways of coupling, e.g., through the fluid of the cochlea, or simply through local interactions through tissue or the tectorial membrane (e.g. [50]), but as yet there are no models that compare their likely effects on SOAE.

**Fig. 2:**
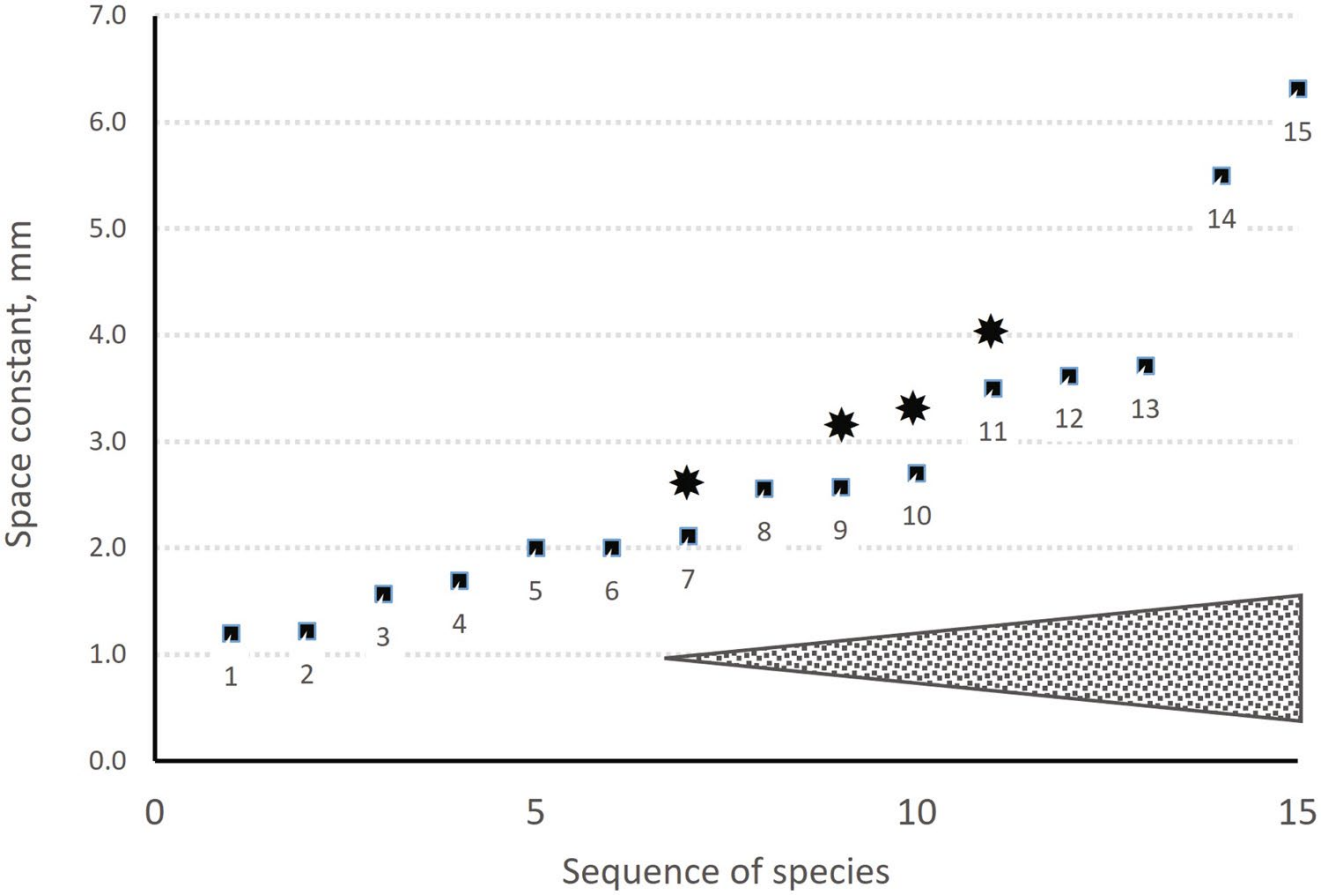
Space constants in mm/octave along the hearing organ in the same sequence as the mammalian species shown in Table 1. Note that the hearing range for the Dromedary is not known. Species known to demonstrate SOAE are indicated by an asterisk. The widening arrowhead at the bottom right reflects the hypothesis that SOAE are more and more likely to occur in species that have space constants higher than ~ 2 mm/octave. The numbers at each data point indicate the species’ numbers as given in the first column of Table 1; asterisks denote species with confirmed SOAE. The frequency response ranges of some of these species are given by Heffner and Heffner [65], their Fig. 2. Note that the mouse does not have an asterisk, since only one single SOAE with 1dB amplitude has been reported in wild-type mice and no tonal suppression evidence was provided [45]

Since cochlear lengths and auditory ranges are well enough known for many mammalian species, a table comparing these is of interest (Table 1, shown graphically in Fig. 2), especially regarding the production of SOAE and its dependence on mechanical coupling. In Table 1, the coupling strength provided by the tectorial membrane per mm of cochlear length in all mammals is assumed to be the same, so that “coupling” depends more on the physical distance between elements responding to different frequencies. On this basis, it is predicted that SOAE will essentially depend on the frequency space constant being large enough, and in the table the species are organized from the smallest to the largest space constant. It is assumed that SOAE are more likely to be measurable in cochleae in which the space constant exceeds some (still unknown) minimum. The table and Fig. 2 suggest that under this assumption, cochleae with increasingly more than ~ 2 mm per octave are more and more likely to produce SOAE. Cochleae with large space constants are often found in large mammals and unfortunately, most of those species have not yet been adequately studied. One group that should also attract further attention regarding SOAE is that of the constant-frequency bats that have extensive acoustic foveae at very high frequencies. In the mustached bat *Pteronotus parnelli*, for example, Kössl [51] reported two SOAE recorded following stimulation of the bat’s foveal region for SFOAE measurements, suggesting that perhaps the acoustic stimulation had induced these “SOAE”. More data on this question would be very interesting.

**Table 1:** Acoustic and structural data underlying Fig. 2

	Species	Audiogram or map?	SOAE?	Octaves	O of Corti length, mm	Space constant mm/oct	Source of data
1	Mouse	m		5	6	1.20	[9]
2	Gerbil	m		9	11	1.22	[10]
3	Mole rat Fukomys	m		7	11	1.57	[11]
4	Rat	m		6.5	11	1.69	[12, 52]
5	Mole rat Spalax	m		6.5	13	2.00	[11, 53]
6	Squirrel monkey	a		9.5	19	2.00	[54, 55]
7	Guinea pig	m	*	9	19	2.11	[56, 57]
8	Cat	m		9	23	2.56	[58, 59]
9	Macaque	a	*	10.5	27	2.57	[60, 61]
10	Chinchilla	m	*	8.5	23	2.71	[62]
11	Human	m	*	10	35	3.50	[49, 58]
12	Cow	a		10.5	38	3.62	[49]
13	Bottlenose dolphin	a		10.5	39	3.71	[49]
14	Cryptomys® fovea	m		1	5.5	5.50	[63]
15	Elephant	a		9.5	60	6.32	[49]
	Dromedary	-		-	40.5	-	[64]

Octave hearing ranges and space constants in different mammalian species were assessed from audiogram or cochlear map data in the publications indicated

An asterisk indicates that SOAE have been reported. ® Now known as *Fukomys*

One example of a large mammal that has received some attention is the tiger, which has a cochlea that is 35.5 mm long and thus much longer than that of the cat [66]. From what we know about its hearing, unlike the domestic cat, the tiger also has excellent low-frequency, perhaps even infrasound hearing [67, 68]. Such low frequencies occupy large extents of the cochlea (e.g. there are 6 octaves between 15Hz and 1 kHz) and we have no cochlear map of the tiger. Even though the upper frequency limit is lower in the tiger than the cat, the difference is less than one octave, and there are thus likely to be 5 more octaves in the tiger cochlea than that of the cat. The space constants may thus not differ between these species. The tiger has thus not been included in Fig. 2. An exception to the assumption of similar intra-cochlear coupling arises when the tectorial-membrane coupling has been reduced in mutant mice (not shown in the table). In addition, in these mutants the tonotopic map is not known and may strongly diverge from those of non-mutant mice.

Of course, in such a complex mechanical system as that of the ear, with its species-specific iterations in the middle and inner ear, the number of components with potential to influence the production and spectral patterns of SOAE is large. Thus, this review only touches on one aspect that shows particular promise [69] of providing an explanation once sufficient data become available. Of course other aspects remain open for detailed consideration.

## Conclusions

In the diverse groups of land vertebrates, comparative studies have shown that different tectorial structures led to unique degrees of coupling that enable similar frequency selectivity even in small hearing organs. In this review it is hypothesized that SOAE arise when the coupling among hair cells is weak enough to enable hair-cell collective activity to emerge from the organ and be measurable outside the middle ear. There are, of course, other factors that may determine SOAE patterns, or even their appearance, such as the generation of standing waves depending on so-called organ of Corti roughness and the strength of middle-ear reflectance. An assessment of the present concept and other models would benefit from more studies on species for which we have little or no data (e.g., the cat), but especially of large land mammals with long cochleae (e.g., cattle, camel or dromedary, elephant), in which the present comparison of space constants would predict a very high likelihood of the presence of SOAE. Perhaps domestication of these species may even make it possible to measure spectra in awake, tame animals!
